# Pharmacological treatment of female sexual dysfunction: a critical analysis of the placebo and nocebo effects

**DOI:** 10.31744/einstein_journal/2020ED5616

**Published:** 2020-06-17

**Authors:** Lucia Alves da Silva Lara

**Affiliations:** 1 Faculdade de Medicina de Ribeirão Preto Universidade de São Paulo Ribeirão PretoSP Brazil Faculdade de Medicina de Ribeirão Preto, Universidade de São Paulo, Ribeirão Preto, SP, Brazil.

The placebo effect rates attributed to the pharmacological treatment of female sexual dysfunctions is 67.7%.^(1)^The placebo effect is beneficial and produced by a substance with no chemical action, which leads to improved response of patients to complaints. The nocebo effect refers to the negative effect of a substance, with or without a chemical action, which worsens the complaints about a disease.^(2)^ These phenomena are known since the 19^th^ century, when placebo obtained the significance of a medication.

The mechanism of placebo and nocebo effects is not well known. The theory is that these effects derive from a biopsychic response to an inert treatment, and such response is associated with past experiences and verbal suggestions that generate positive expectations of improvement, or negative expectations of clinical involvement, which can produce or worsen symptoms.^(2)^

In general, it is a challenge to determine the prevalence of the nocebo effects on pharmacological treatments due to ethical issues related to the induction of this effect. According to a recent review, the few randomized studies that used the nocebo effect were about pain, male sexual dysfunction, and Parkinson’s disease.^(3)^ In the same way, it is challenging to determine the true prevalence of the placebo effect on treatment of sexual dysfunctions, because of the difficulties in isolating the true placebo response of the innumerable biological, psychic, relational, emotional, and environmental variables, among others, which pervade the human sexual response.

The multiple dimensions of the human sexual response mechanism explain the large number of factors related to sexual difficulties that are highly prevalent worldwide. In the Brazilian population, 33% of men and 49% of women^(4)^ report some type of sexual complaint. These numbers vary broadly considering sex, age group, and type of sexual complaint, and the most prevalent complaints are hypoactive sexual desire in women, and erectile dysfunction in men.

The multiplicity of factors involved in sexual response and in reaction of the person to treatments partly justifies the divergent results of clinical trials, which evaluate the placebo response in sexual dysfunctions treatments. Another important obstacle in assessing this effect is the divergence of results relative to the effects of drugs, due to methodological limitations of the studies, which are unable to control all variables responsible for the complexity of sexual dysfunctions diagnosis. Additionally, there are personal characteristics of the subjects, which predispose them to more or less nocebo and placebo effects.

An ancient review highlighted that 19% of healthy people experimented symptoms of headache, weakness, and somnolence due to the placebo effect of inert drugs.^(5)^ On the other hand, a recent review compiled data from eight randomized clinical studies that evaluated 2,236 women who received pharmacological treatment for sexual dysfunctions, including flibanserine, bupropion, botulinum toxin, intravaginal prasterone, oxitocin, ospemifen, and bremelanotide. The intervention group had a 5.35-fold increment in the total score of the Female Sex Function Index (FSFI), while 1,723 women who received placebo had a 3.62-fold increase in the total FSFI score.^(1)^In this study, the placebo effect accounted for two thirds of improvement in sexual complaints. The methodology of these studies is not always comparable, but this percentage of placebo response has contributed towards hampering the approval of drugs for the treatment of female sexual dysfunctions.

## Factors that influence the placebo and nocebo effects

These effects are influenced by external factors or environmental, in addition to internal or personal factors. The cascade of events that trigger the nocebo and placebo responses begins by internal commands and suggestions of the mind, and advances as the person develops these commands, which can transform into signs and symptoms of a disease.

The emotional status, personal and humor characteristics, cognition and personality of the individual, and female sex – which has a greater tendency towards the placebo effect as compared to males – among others, are crucial for the activation of these mechanisms and influence the genesis of the diseases and the response to treatment.^(3)^ An example of this is the nocebo effect promoted by negative media information about a given medication with known beneficial action. In New Zealand, a negative advertisement on television about a drug known for its effectivity, such as levothyroxine, led to an increase in adverse events related to this medicine.^(6)^

The negative expectation of the person or the negative experiences of patients relative to an intervention can exert a nocebo effect, leading to reduced efficacy of clinical interventions that could be beneficial.^(3)^The nocebo effect is also identified in the genesis of diseases. For example, hopelessness is an important component in the creation of depression; hypochondria and conversion are associated with an expectation of having a disease; anxiety disorders and panic syndromes may be associated with people who feed on catastrophic thoughts of the eminence of disease or death.^(3)^

The placebo effect can also be influenced by the type of intervention, and those involving the use of devices promote more the placebo effect in comparison with the purely pharmacological interventions.^(7)^

## Mechanism of placebo and nocebo effects

The mechanism of these effects is yet unknown, but theories point to a possible connection with the individual’s conditioning system and expectations. The conditioning model follows the principles of Pavlov’s experiments,^(8)^ according to which, an active substance causes a beneficial effect, and when replaced by an inert substance with the same characteristics of color, smell, appearance and size, this inactive compound will continue to exert the same effect of the active substance. This response is associated to an activation of the recompense system, in which the activation of cortical neurons results in the excitatory stimuli of glutamate that activate dopaminergic neurons,^(9)^ thus promoting the sensation of well-being.

The model of expectations considers that thoughts and beliefs can influence human neurobiology creating a therapeutic process.^(10)^ This model also may explain the mechanism of the nocebo effect where the negative expectations activate the hypothalamic-pituitary-adrenal (HPA) axis, stimulating the production of the adrenocorticotropic hormone (ACTH) and cortisol, which promote the mechanism of anticipatory anxiety.^(11)^ Simultaneously, both ACTH and cortisol activate the cholecystokinin system, which is responsible for regulating nociception, anxiety, and memory. The memory is also activated by the conditioning mechanism. It is important to point out that expectation is an individual dimension, that is under the influence of individual values, culture, beliefs, and myths. Thus, the symptoms of physical and psychic dysfunction promoted by activation of the HPA are provaded to the personal susceptibility of each individual.

## True dimension of the placebo and nocebo effects in the pharmacological treatment

To define the true dimension of the placebo and nocebo effects on pharmacological sexual dysfunctions is a challenge, due to multiple confounding factors that should be taken into account when evaluating these effects. As is widely known, sexual dysfunctions have a close association with the person’s psychic condition, which can influence the conditioning systems and expectations related to the placebo effect. Moreover, there are variations in the personality of people that make them salutogenic or pathogenic, pharmacophilic or pharmacophobic, and presenting more or less suggestibility to negative or positive aspects. There are also people who foster hope or hopelessness, and patients prone to trust medical interventions, while others are more skeptical; those who are optimist or a pessimist; and those who are self-confident and predisposed to new sensations. These and so many other personal characteristics influence the psychological component in the process of disease and cure, as well as the physiological mechanisms of the response to pharmacological intervention.^(12)^

The assessment of the placebo effect in studies is hard due to the common lack of diagnostic criteria for sexual dysfunctions. An example is the misuse of the term “libido”, when one intends to evaluate sexual desire, or the inappropriate use of “sexual pleasure”, when the purpose is to evaluate the sexual satisfaction. Another point is that randomized studies do not always use an adequate tool to assess the female sexual response. Also, the terminology used to report results is not always adequate and consistent with the instrument used.^(13)^ Many studies use as primary outcome the term “sexual frequency”, which, *per se*, cannot be used as a marker of the improvement of the sexual function. In addition, the number of penis-vagina relations or sexual encounters is not solely, a marker of a woman’s sexual satisfaction.^(14)^ The measurement of female sexual satisfaction involves the use of specific questionnaires for this end. It is important to remember that the worldwide used “Female Sexual Dysfunction Index” comprises constructs that evaluate the phases of female sexual response^(15)^ but, there is a need for more accurate questionnaire to evaluate the woman’s sexual satisfaction. Therefore, it is limited tools to assess the effectiveness of a pharmacological treatment for female sexual dysfunction. These types of “dystocia” in the inappropriate use of terminologies to characterize sexual dysfunctions and the inappropriate evaluation of the sexual response´s phases may lead to misresults.

In summary, considering all the cofounding variables that permeate the placebo and nocebo effects, it is challenging to separate them from the true pharmacological effect of the drugs used to treat female sexual dysfunctions. Therefore, we concluded that the evidence on this theme is weak due to methodological limitations of the studies, such as the unsuitable use of questionnaires that are unable to achieve the objectives of the studies, the misuse of terms that are not criteria to define sexual dysfunctions, and the lack of criteria to characterize the sexual complaint. In this way, caution is recommended to attribute 67% of placebo effect to the pharmacological treatment of female sexual dysfunctions.

For future studies, we recommend the use of validated questionnaires that evaluate all phases of the female sexual response, including sexual satisfaction. We also recommend the use of adequate terminology and of defined criteria to characterize sexual dysfunctions. These actions contribute towards the control of countless variables implied in the human sexual response, in order to determine the true effect of pharmacological treatment in male and female sexual dysfunctions.
