# Supplementary material for: Pharmacological treatment of female sexual dysfunction: a critical analysis of the placebo and nocebo effects
Source: Einstein (Sao Paulo). 2020 Jun 17;18:eED5616. doi: 10.31744/einstein_journal/2020ED5616 (PMC7299526; doi:10.31744/einstein_journal/2020ED5616)
Supplement: Translation

# Translation: Tratamento farmacológico das disfunções sexuais femininas: uma análise crítica dos efeitos placebo e nocebo

**Authors:** Lucia Alves da Silva Lara

**Affiliations:** 1 Brasil Faculdade de Medicina de Ribeirão Preto, Universidade de São Paulo, Ribeirão Preto, SP, Brasil.

O valor atribuído ao efeito placebo no tratamento farmacológico das disfunções sexuais femininas é de 67,7%.^(1)^ O efeito placebo é benéfico e produzido por uma substância sem ação química, que provoca melhora da resposta do paciente às queixas. Já o efeito nocebo refere-se ao efeito negativo de uma substância, com ou sem ação química, que piora as queixas referentes a uma doença.^(2)^ São fenômenos reconhecidos a partir do século 19, quando o placebo obteve o significado de medicação.

O mecanismo dos efeitos placebo e nocebo não é bem conhecido. A teoria é a de que esses efeitos derivam de uma resposta biopsíquica a um tratamento inerte, e tal resposta associa-se a experiências passadas e a sugestões verbais, que geram expectativas positivas de melhora, ou negativas de comprometimento clínico, que podem produzir ou piorar sintomas.^(2)^

Em geral, é desafiador determinar a prevalência do efeito nocebo nos tratamentos farmacológicos devido às questões éticas relacionadas à indução desse efeito. De acordo com revisão recente, os poucos estudos randomizados que utilizam efeito nocebo são sobre a dor, função sexual masculina e doença de Parkinson.^(3)^ Da mesma forma, é um desafio determinar a real prevalência do efeito placebo no tratamento das disfunções sexuais, pelas dificuldades para isolar a verdadeira resposta placebo das inúmeras variáveis biológicas, psíquicas, relacionais, emocionais, ambientais, entre outras, que permeiam a resposta sexual humana.

As múltiplas dimensões do mecanismo da resposta sexual humana explicam o grande número de fatores relacionados com as dificuldades sexuais que são altamente prevalentes em todo o mundo. Na população brasileira, 33% dos homens e 49% das mulheres^(4)^ referem algum tipo de queixa sexual. Esses números variam, amplamente, com o sexo, a faixa etária e o tipo de queixa sexual, sendo o desejo sexual hipoativo, em mulheres, e a disfunção erétil, nos homens, as queixas mais prevalentes.

A multiplicidade de fatores envolvidos na resposta sexual e na reação da pessoa aos tratamentos responde, em parte, pela divergência dos resultados dos ensaios clínicos que avaliam a resposta placebo no tratamento das disfunções sexuais. Outro importante entrave na apreciação desse efeito é a divergência dos resultados em relação aos efeitos das drogas, por conta das limitações metodológicas dos estudos, que não conseguem controlar todas as variáveis responsáveis pela complexidade do diagnóstico das disfunções sexuais. Além disso, existem características pessoais dos sujeitos mais ou menos predispostos aos efeitos nocebo e placebo.

Uma revisão antiga evidenciou que 19% de pessoas saudáveis experimentaram o efeito placebo de drogas inertes, com sintomas como cefaleia, fraqueza e sonolência.^(5)^ Já revisão recente compilou os dados de oito estudos clínicos randomizados que avaliaram 2.236 mulheres que receberam tratamento farmacológico para disfunções sexuais, incluindo flibanserina, bupropiona, toxina botulínica, prasterona intravaginal, oxitocina, ospemifeno e bremelanotide. Em conjunto, esse grupo teve um incremento de 5,35 vezes no escore total do Índice de Função Sexual Feminina (IFSF), enquanto as 1.723 mulheres que receberam placebo tiveram incremento de 3,62 vezes no escore total do IFSF.^(1)^ Nesse estudo, o efeito placebo respondeu por dois terços da melhora nas queixas sexuais. Nem sempre a metodologia desses estudos é comparável, mesmo assim, esse percentual de resposta placebo tem contribuído para dificultar a aprovação de medicamentos para o tratamento das disfunções sexuais femininas.

## Fatores que influenciam nos efeitos placebo e nocebo

Esses efeitos são influenciados por fatores externos ou ambientais, além dos internos ou pessoais. A cascata de eventos que desencadeia as respostas nocebo e placebo começa por comandos internos e sugestões da mente, e avança conforme a pessoa elabora esses comandos, os quais podem se transformar em sintomas e sinais de uma doença.

O estado emocional, as características pessoais e de humor, a cognição, a personalidade, o sexo feminino – que tem mais propensão à resposta placebo em relação aos homens – , entre outros, são cruciais para a ativação desses mecanismos e influenciam, tanto na gênese das doenças como na resposta ao tratamento.^(3)^ Um exemplo disso é o efeito nocebo promovido por informações midiáticas negativas sobre um determinado medicamento com ação benéfica reconhecida. Na Nova Zelândia, uma propaganda negativa na televisão sobre uma droga reconhecidamente efetiva, como a levotiroxina levou a um incremento no número de efeitos adversos relacionados a essa droga.^(6)^

As expectativas negativas da pessoa ou as experiências negativas de pacientes em relação a uma intervenção podem exercer um efeito nocebo, levando à redução da eficácia de intervenções clínicas que poderiam ser benéficas.^(3)^ O efeito nocebo também é identificado na gênese de doenças. Por exemplo, a desesperança é um componente importante na gênese da depressão; a hipocondria e a conversão estão associadas a expectativa de ter uma doença; o transtorno de ansiedade e a síndrome do pânico podem estar associados a pessoas que nutrem pensamentos catastróficos de eminência de doença ou morte.^(3)^

O efeito placebo também pode sofrer influência do tipo de intervenção, e as que envolvem a utilização de aparelhos promovem mais o efeito placebo em comparação às intervenções puramente farmacológicas.^(7)^

## Mecanismo dos efeitos placebo e nocebo

O mecanismo desses efeitos ainda é desconhecido, porém as teorias apontam para possível ligação ao sistema de condicionamento e às expectativas da pessoa. O modelo de condicionamento segue os princípios dos experimentos de Pavlov,^(8)^ segundo o qual uma substância ativa provoca um efeito benéfico e, quando substituida por uma substância inativa contendo as mesmas características em relação a cor, cheiro, aspecto e tamanho, continua exercendo o efeito provocado pela substância ativa. Essa resposta está relacionada com a ativação do sistema de recompensa, no qual a ativação de neurônios corticais resulta em estímulos excitatórios glutamaltérgicos que ativam neurônios dopaminérgicos,^(9)^ promovendo a sensação de bem-estar.

O modelo de expectativas considera que os pensamentos e crenças podem influenciar na neurobiologia humana, a ponto de criar um processo terapêutico.^(10)^Esse modelo serve, também, para explicar o mecanismo do efeito nocebo, ou seja, as expectativas negativas ativam o eixo hipotálamo-hipófise-adrenal (EHH) estimulando a produção do hormônio adrenocorticotrófico e do cortisol, que promovem o mecanismo de ansiedade antecipatória.^(11)^ Ao mesmo tempo, promove a ativação do sistema de colecistocinina, responsável por regular a nocicepção, a ansiedade e a memória, e esta é também ativada pelo mecanismo de condicionamento. Vale lembrar que a expectativa é uma dimensão individual, sujeita aos valores individuais, à cultura, às crenças e aos mitos, e os sintomas de disfunção física e psíquica promovidos pela ativação do EHH são proporcionais à suscetibilidade de cada indivíduo.

## Dimensão real dos efeitos placebo e nocebo no tratamento farmacológico

Definir a dimensão real dos efeitos placebo e nocebo nos tratamentos farmacológicos das disfunções sexuais é um desafio por conta dos múltiplos fatores confundidores, que devem ser levados em conta na avaliação desses efeitos. Como são largamente conhecidas, as disfunções sexuais têm estreita associação com a condição psíquica da pessoa, o que pode influenciar nos sistemas de condicionamento e nas expectativas, relacionados com o efeito placebo. Associado a isso, existem variações na personalidade das pessoas que as tornam mais salutogênicas ou patogênicas, farmacofílicas ou farmacofóbicas, e mais ou menos sugestionáveis para o negativo ou o positivo. Existem, ainda, as pessoas que nutrem a esperança e as que fomentam a desesperança, e pacientes propensas a confiarem nas intervenções médicas e nos médicos, enquanto outras são mais céticas; as que são otimistas e as pessimistas; e as que são autoconfiantes e predispostas às novas sensações. São essas e outras tantas características pessoais que influenciam no componente psicológico do processo da doença e da cura, bem como nos mecanismos fisiológicos da resposta a uma intervenção farmacológica.^(12)^

Na avaliação do efeito placebo nos estudos, é também bastante comum se deparar com dificuldades relacionadas à falta de critérios para o diagnóstico das disfunções sexuais. Um exemplo é o uso equivocado do termo “libido”, quando se pretende avaliar o desejo sexual, ou do termo “prazer sexual”, quando se pretende avaliar a satisfação sexual. Outro ponto é que, nem sempre, os estudos randomizados utilizam instrumento adequado para avaliar a resposta sexual feminina, e nem a terminologia utilizada para demonstrar os resultados é adequada e coerente com o instrumento que utiliza.^(13)^ Muitos estudos utilizam como desfecho primário a “frequência sexual” que, por si só, não pode ser utilizada como marcador de melhora da função sexual. No entanto, o número de relação pênis-vagina ou de encontros sexuais, isoladamente, não são marcadores de satisfação sexual da mulher.^(14)^ Isso porque, aferir a satisfação sexual feminina envolve a utilização de questionários específicos para esse fim. Vale lembrar que o consagrado e mundialmente utilizado *Female Sexual Disfunction Index* compreende construtos que avaliam as fases da resposta sexual feminina,^(15)^ porém, isoladamente, é um questionário limitado para avaliar a efetividade de um tratamento farmacológico para a disfunção sexual feminina. Essas “distócias” no uso inadequado de terminologias para caracterizar as disfunções sexuais e a avaliação inadequada das fases da resposta sexual, mesmo usando questionários validados, levam a resultados equivocados.

Em resumo, considerando todas as variáveis confundidoras que permeiam os efeitos placebo e nocebo, é um desafio separar tais resultados do efeito farmacológico real dos medicamentos utilizados para o tratamento das disfunções sexuais femininas. Portanto, pode-se concluir que a evidência sobre essa temática é fraca na maioria dos estudos que apresentam limitações metodológicas, que incluem o uso inadequado de questionários nem sempre adequados para responder os objetivos dos estudos, a utilização indevida de terminologias que não são mensuráveis para a classificação das disfunções sexuais e a falta de critérios para caracterizar a queixa sexual. Dessa forma, é recomendada cautela para atribuir, de fato, 67% de efeito placebo no tratamento farmacológico das disfunções sexuais femininas.

Para estudos futuros, recomenda-se o uso de questionários, validados que avaliem todas as fases da resposta sexual feminina incluindo a satisfação sexual. É recomendado, também, o uso de terminologia adequada e de critérios definidos para caracterizar as disfunções sexuais. Essas ações contribuem para controlar as inúmeras variáveis implicadas na resposta sexual humana, a fim de determinar o real efeito do tratamento farmacológico nas disfunções sexuais em homens e mulheres.
